# Elevated neutrophil to lymphocyte ratio as an indicator of secondary erythema nodosum, a retrospective observational study

**DOI:** 10.3906/sag-1810-3

**Published:** 2019-04-18

**Authors:** Yıldız HAYRAN, Ayşe ÖKTEM, Buket ŞAHİN, Pınar İNCEL UYSAL, Nuran ALLI, Başak YALÇIN

**Affiliations:** 1 Department of Dermatology, Ankara Numune Training and Research Hospital, Ankara Turkey

**Keywords:** Erythema nodosum, neutrophil to lymphocyte ratio, etiological factors

## Abstract

**Background/aim:**

Erythema nodosum (EN) is an inflammatory disorder of subcutaneous tissue. Although etiopathogenesis of the disease is unknown, many predisposing factors such as infections, systemic disease, and drugs have been identified. Neutrophil to lymphocyte ratio (NLR) has been shown to be a novel inflammatory marker in many dermatological diseases. The aim of our study is to investigate NLR in EN patients and evaluate its relation to the underlying cause of the disease.

**Materials and methods:**

Between 2014 and 2018, clinical and laboratory data of 395 patients diagnosed with EN and 395 controls were extracted from patient files. EN patients were grouped as idiopathic EN and secondary EN (EN with an identified underlying cause). Clinical and laboratory characteristics of the two groups were compared.

**Results:**

NLR was elevated in EN patients compared to controls (median of 2.38 vs. 1.55, P < 0.001). Among EN patients, NLR was also elevated in patients with secondary EN. In multivariate logistic regression model NLR (> 2.11), RDW-CV (> 13.65), and CRP (> 5.5) were identified as risk factors for secondary EN (relative risks were 17.16, 2.69, and 2, respectively).

**Conclusion:**

Elevated NLR (> 2.11) may be used as a parameter to discriminate secondary EN from idiopathic EN.

## 1. Introduction 

Erythema nodosum (EN) is an inflammatory disorder of subcutaneous tissue characterized by erythematous, tender subcutaneous nodules predominantly affecting lower extremities. The pathogenesis of EN is not well understood. Hypersensitivity reaction against an unknown antigen is the main theory, but some authors suggested that neutrophils also contribute to EN pathogenesis (1,2). Kunz et al. showed that reactivated neutrophils increased in patients with EN and the ratio of reactive oxygen intermediates producing polymorphonuclear neutrophils correlate with disease severity (3). 

Although one third of EN is idiopathic, more than half of the patients have secondary EN with etiologic factors such as infections, systemic disease, and drugs that support the hypersensitivity reaction hypothesis in pathogenesis (4–6). Identifying and eliminating these etiological factors is the first step of EN treatment and may limit the recurrences of EN (1,7–9). Medical history and physical examination easily identify known infections and systemic diseases, but in patients with inconclusive history additional laboratory tests are needed. Although some demographic, clinical, and laboratory features such as advanced age, atypical localization of the lesions, increased ESR and CRP are more common in patients with secondary EN, no laboratory parameter with high sensitivity and specificity was identified to recognize EN patients with a possible precipitating factor. 

Neutrophil to lymphocyte ratio (NLR) is a novel inflammatory marker identified in cardiac and inflammatory disorders (10–13). NLR increases in inflammatory diseases, correlates with disease severity and conventional inflammatory markers, and may predict response to treatment and survival (14–20). The aim of this study is to evaluate NLR in EN patients, investigate its relationship with etiological factors and its value as a predictor of secondary EN. 

## 2. Materials and methods

### 2.1. Study design and data source

**Figure 1:**
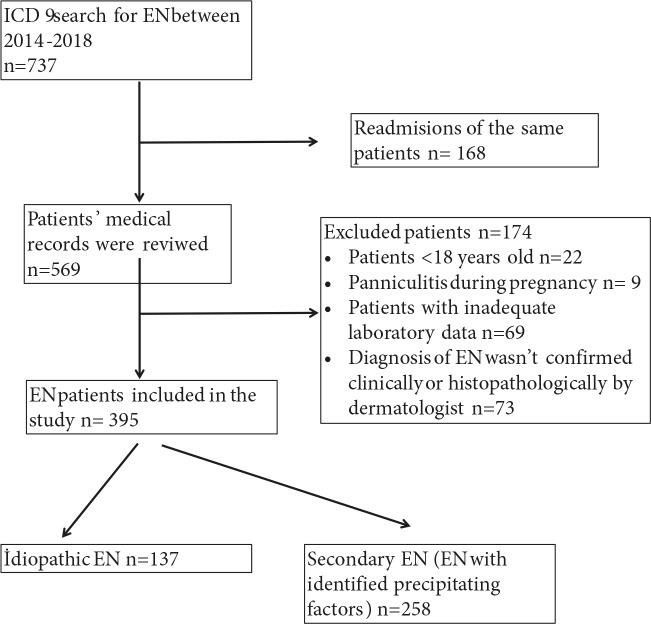
Flowchart summarizing patient inclusion and exclusion processes.

We retrospectively analyzed patients diagnosed with EN from January 2014 to January 2018 in a single tertiary referral center using International Classification of Diseases, Ninth Revision codes. A dermatologist reviewed patient charts from a local hospital database and dermatology department’s archive of 737 patients. After elimination of unfit records (Figure 1), 395 patients diagnosed with EN were included in this study. The study was approved by the Ethics Committee of Ankara Numune Training and Research Hospital (E–18–1814).

### 2.2. Study population 

Demographic, clinical, and laboratory data including age, sex, cause of EN, presence of a previous EN attack, complete blood count with differential, erythrocyte sedimentation rate (ESR), C-reactive protein (CRP), and skin biopsy results were retrieved from patient records. All patients included in the study were tested for upper respiratory tract infection (URTI) using antistreptolysin-O (ASO) and throat culture, for urinary tract infection (UTI) using complete urinary analysis and urine culture, and for rheumatologic diseases using antinuclear antibodies, extractable nuclear antigen, rheumatoid factor, anti-ds DNA, antigliadin, antitransglutaminase antibody, and antiendomysium. Screening for other etiological factors was performed based on patient history. Drug use within 3 weeks prior to EN was recorded. Antibiotic use was not included in drug induced EN but classified within infection related EN. EN patients were divided into two major groups as idiopathic EN and secondary EN according to the presence of an etiological factor. Secondary EN patients were further divided into two groups as (i) secondary EN patients with a precipitating factor either initially known at admission due to a prior diagnosis or recognized in an overt symptomatic presentation, and (ii) those diagnosed after detailed laboratory workup. 

Same number (n = 395) of age and sex matched noninflammatory, noninfective dermatosis patients from the same period were included in the study as control group. 

### 2.3. Laboratory data

In all cases, complete blood count with differential, ESR (millimeters (mm)/hour), and CRP (mg/dL) were recorded from patient files. NLR, monocyte-lymphocyte ratio (MLR), and platelet-lymphocyte ratio (PLR) were calculated by dividing the absolute neutrophil, monocytes, and platelet counts by the absolute lymphocyte counts, respectively: NLR = neutrophils (10³ µL)/lymphocytes (10³ µL), MLR = monocytes (10³ µL)/lymphocytes (10³ µL), PLR = platelets (10³ µL)/lymphocytes (10³ µL). 

### 2.4. Statistical analysis

All statistical analyses were performed using IBM SPSS Statistics for Windows, Version 21.0 (IBM Corp., NY, USA). Quantitative and qualitative variables were analyzed using Chi-square test and Mann-Whitney U test. Variables with a P value < 0.2 in univariate analysis were further entered into logistic regression analysis to determine the independent predictors of secondary EN. Spearman’s correlation test was used for correlation analysis of quantitative variables and a P value < 0.05 was considered as statistically significant.

## 3. Results

### 3.1. Patient characteristics 

Eighty percent of the patients were female (n = 316) and 20% were male (n = 79). The median age of patients was 39 (IQR: 29–51). The main characteristics of EN patients are summarized in Table 1. Among the EN patients, 34.7% (n = 137) were idiopathic and 65.3% (n = 258) had a secondary factor that may cause, precipitate, or aggravate EN. Among 258 secondary EN patients, 56.6% (n = 146) had infection, 33.7% (n = 87) had systemic diseases, 6.6% (n = 17) had drug use, and 3.1% (n = 8) had malignancy as a cause. Infections, systemic diseases, and drugs are listed in detail in Table 2. The cause of secondary EN was initially known in 40.3% (n = 104) of the patients. After routine screening, 56.8% of patients with infection and 81.6% of patients with systemic diseases were identified (Table 3). All of the malignancies and drugs reported by the patients were listed as participating factors in medical histories. 

**Table 1:** Characteristics of EN patients.

Characteristic	N (%)
Age (years)	39 (29–51) *
Sex, male	79 (20)
EN type Idiopathic EN Secondary EN	137 (34.7)258 (65.3)
RecurrencePresentAbsent	64 (16.2)331 (83.8)
Diagnosis Histopathological Clinical	78 (19.7)352 (80.3)

* Median (IQR), EN: erythema nodosum.

**Table 2:** Causes of secondary EN.

Cause	N (%)
Infection URTI UTI Skin LRTI GIS	146 (56.6)82 48533
Systemic diseases Rheumatologic Pulmonary Intestinal	87 (33.7)71106
Drug OCs NSAIDs	17 (6.6) 12 5
Malignancy Hematological malignancies Endometrial cancer Breast cancer	8 (3.1)521
Total	258 (100)

EN: erythema nodosum, URTI: upper respiratory tract infection; UTI: urinary tract infection, LRTI: lower respiratory tract infection, GIS: gastrointestinal system, NSAIDs: nonsteroidal antiinflammatory drugs, OCs: oral contraceptives.

**Table 3:** Etiological factors in secondary EN patients by time of discovery.

Cause	Known atadmission N (%)	Diagnosed afterwork-up N (%)	Total
Infection UTI URTI Other	63 (43.2)24813	83 (56.8)46343	146 (56.6)
Systemic diseases Rheumatologic Pulmonary Other	16 (18.4)1213	71 (81.6)5993	87 (33.7)
Drug OCs NSAIDs	17 (100)125	-	17 (6.6)
Malignancy Hematological malignancies Endometrial cancer Breast cancer	8 (100)521	-	8 (3.1)
Total	104 (40.3)	154 (59.7)	258 (100)

URTI: upper respiratory tract infection, UTI: urinary tract infection, LRTI: lower respiratory tract infection, GIS: gastrointestinal system, NSAIDs: nonsteroidal antiinflammatory drugs, OCs: oral contraceptives.

Recurrent EN was observed in 16.2% of the patients, while in 83.8% of the patients no previous history of EN was noted. Age, percentage of secondary EN, and RDV-CV levels were higher in patients with recurrent EN compared to nonrecurring EN (P = 0.044, 0.001, and 0.042). Although CRP, NLR, and MLR levels were also elevated, the difference was not statistically significant (P = 0.054, 0.15, and 0.19, respectively). Clinical and laboratory features of recurrent and nonrecurrent EN are summarized in Table 4.

**Table 4:** Clinical and laboratory features of recurrent and nonrecurrent EN.

	Nonrecurrent EN	Recurrent EN	P
Age median (IQR)	38 (29–50)	45 (34–51)	0.044
Sex (male/female)	68/263	11/53	0.54
Cause (idiopathic/secondary)	127/204	10/54	< 0.001
Neutrophil	8.05 (6.8–9.4)	8.4 (6.9–9.97)	0.12
MCH	28 (26.4–29.5)	27.3 (25.6–28.8)	0.013
MCHC	32.9 (32–33.7)	32.65 (31.42–33.22)	0.008
RDW-CV	13.7 (13.1–14.7)	14.25 (13.2–15.25)	0.042
CRP	6 (2–18)	10 (4–23)	0.054
NLR	2.31 (1.7–3.52)	2.64 (2.04–3.71)	0.15
MLR	0.25 (0.19–0.36)	0.28 (0.23–0.38)	0.19

IQR: interquartile range, MCH: mean cell hemoglobin, MCHC: mean cell hemoglobin concentration, NLR: neutrophil-lymphocyte ratio, MLR: monocyte-lymphocyte ratio, P: significance level, RDW-CV: red cell distribution width coefficient of variation, CRP: C reactive protein.

In 352 patients EN was diagnosed clinically, and in 78 patients clinical diagnosis was confirmed histopathologically. Both clinically and histopathologically diagnosed EN patients were similar in age and sex (P = 0.39 and P = 0.68), but secondary EN and recurrence was more frequent in histopathologically diagnosed EN (both P < 0.01). 

### 3.2. Laboratory data 

**Figure 2:**
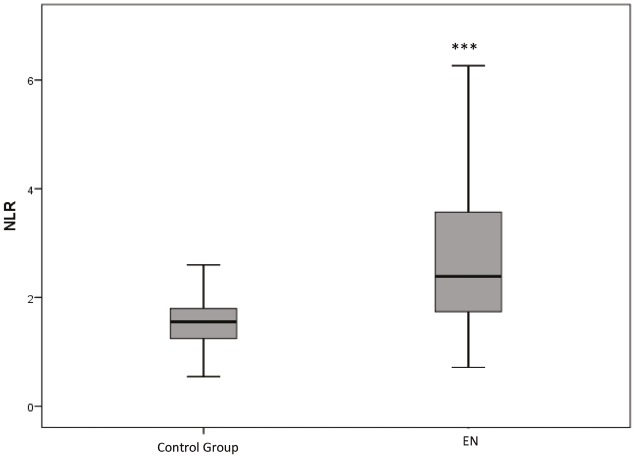
Median NLR was significantly higher in EN patients. *** P < 0.001.

**Figure 3:**
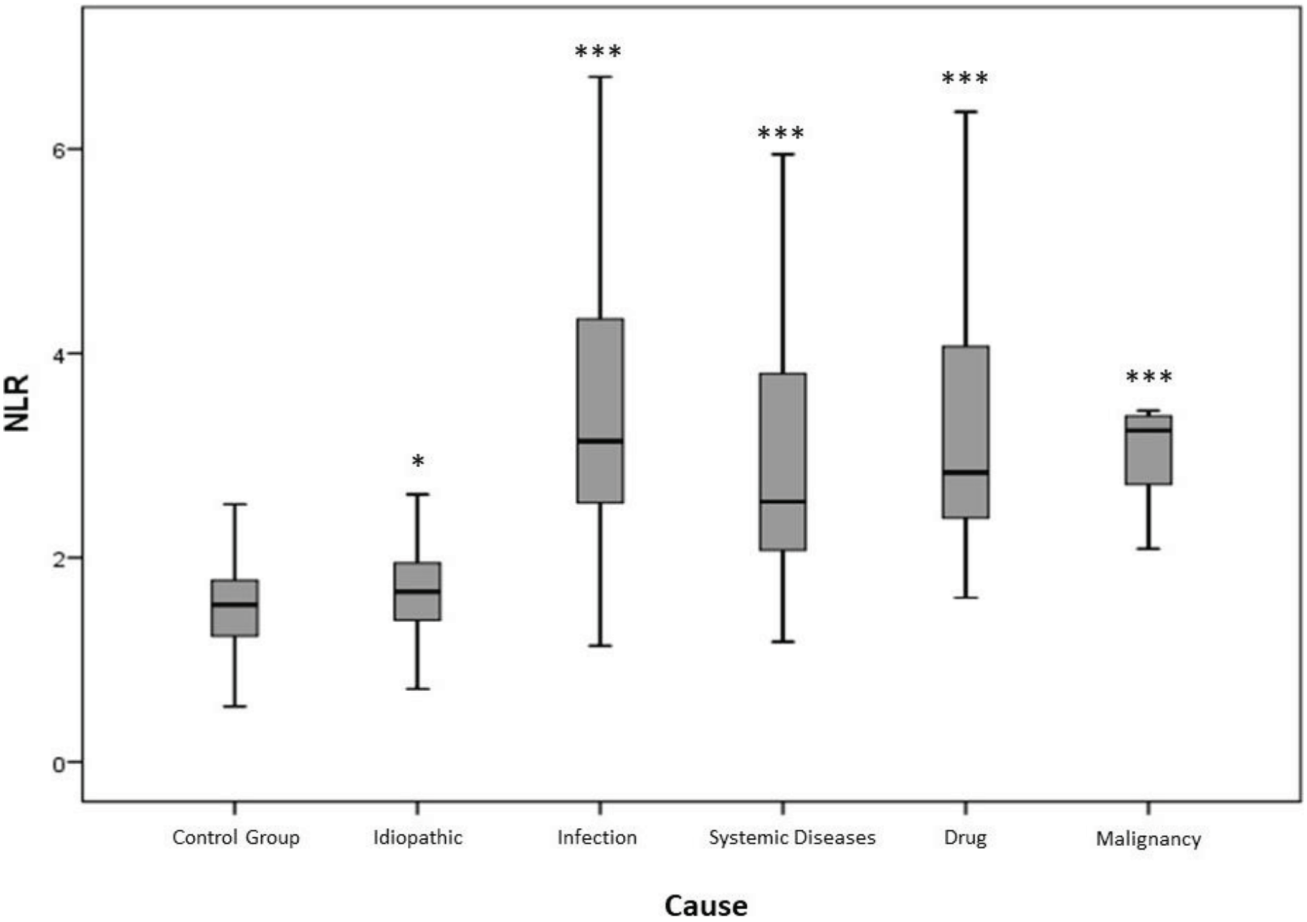
NLR of secondary EN patients was higher than that of idiopathic EN and control group. * P < 0.01, *** P < 0.001.

Laboratory features of EN patients compared to control group are listed in Table 5 and laboratory features of secondary EN patients compared to idiopathic EN are listed in Table 6. NLR was higher in EN patients compared to control group (P < 0.001). Median NLR was 2.38 (IQR 1.73–3.58) in patients and 1.55 (IQR 1.23–1.8) in controls (Figure 2). NLR of both secondary and idiopathic EN patients was significantly higher than control group (P < 0.001 and P = 0.003, respectively) and NLR of secondary EN patients was higher than idiopathic EN (P < 0.001) (Figure 3). 

**Table 5:** Laboratory features of EN patients compared to control group.

	EN patients(n = 395)	Control group(n = 395)	P value
Age (IQR)	39 (30–50)	40 (27–52)	0.45
Sex (male/female)	79/316	79/316	1
WBC (109/L) (IQR)	8.1 (6.87–9.42)	7.2 (6.1–8.4)	<0.001
Neutrophil (109/L) (IQR)	5.05 (3.9–6.5)	3.8 (3.2–4.7)	<0.001
Lymphocyte (109/L) (IQR)	2.1 (1.6–2.52)	2.5 (2.1–3)	<0.001
Monocyte (109/L) (IQR)	0.6 (0.4–0.7)	0.5 (0.4–0.7)	0.02
Hemoglobin (g/dL) (IQR)	13.1 (12.1–14.2)	13.8 (12.9–14.8)	<0.001
MCV (fL) (IQR)	85 (81.27–87.92)	86.3 (83.6–89.1)	<0.001
MCH (pg) (IQR)	27.9 (26.3–29.4)	28.6 (27.2–29.5)	<0.001
MCHC (g/dL) (IQR)	32.8 (31.9–33.6)	32.9 (32.1–33.8)	0.18
Platelet (109/L) (IQR)	280.5 (232.7–332.5)	274 (236–320)	0.23
MPV (fL) (IQR)	10 (8–11)	11 (10–11)	<0.001
PDW (%) (IQR)	16 (13–16.6)	12.3 (10.9–13.72)	<0.001
RDW-CV (%) (IQR)	13.8 (13.1–14.8)	13.1 (12.6–13.7)	<0.001
NLR (IQR)	2.38 (1.73–3.58)	1.55 (1.23–1.8)	<0.001
MLR (IQR)	0.26 (0.2–0.36)	0.21 (0.17–0.25)	<0.001
PLR (IQR)	136.27 (103.47–169.51)	107.6 (87.77–132.22)	<0.001

N: sample size, MCV: mean corpuscular volume, MCH: mean cell hemoglobin, MCHC: mean cell hemoglobin concentration, MPV: mean platelet volume, NLR: neutrophil-lymphocyte ratio, MLR: monocyte-lymphocyte ratio, PLR: platelet-lymphocyte ratio, WBC: white blood cell, P: significance level, PDW: platelet distribution width, RDW-CV: red cell distribution width coefficient of variation.

**Table 6:** Laboratory features of secondary EN patients compared to idiopathic EN.

	Secondary EN(n = 258)	Idiopathic EN (n = 137)	P value
Age (IQR)	38 (29.7–49)	42 (31.5–53)	0.062
Sex (male/female)	52/206	27/110	0.91
WBC (109/L) (IQR)	8.5 (7.1–10.35)	7.3 (6.15–8.45)	<0.001
Neutrophil (109/L) (IQR)	5.6 (4.6–7.3)	3.9 (3.4–4.9)	<0.001
Lymphocyte (109/L) (IQR)	1.9 (1.55–2.4)	2.4 (2–2.8)	<0.001
Monocyte (109/L) (IQR)	0.6 (0.5–0.8)	0.5 (0.4–0.6)	0.01
Hemoglobin (g/dL) (IQR)	12.8 (11.85–13.8)	13.6 (12.5–14.4)	<0.001
MCV (fL) (IQR)	84.8 (80.4–87.55)	85.5 (82.35–88.4)	0.034
MCH (pg) (IQR)	27.6 (26–29.2)	28.3 (26.7–29.7)	0.011
MCHC (g/dL) (IQR)	32.7 (31.8–33.5)	33 (32.1–33.85)	0.050
Platelet (109/L) (IQR)	284 (237.5–345)	275 (226.5–308.5)	0.012
MPV (fL) (IQR)	9 (8–10)	10 (9–11)	0.006
PDW (%) (IQR)	16.2 (13.2–16.7)	14.65 (12.62–16.5)	0.027
RDW-CV (%) (IQR)	13.9 (13.2–15)	13.5 (13.1–14.5)	0.013
NLR (IQR)	2.88 (2.31–4.07)	1.66 (1.39–1.95)	<0.001
MLR (IQR)	0.3 (0.22–0.4)	0.21 (0.17–0.27)	<0.001
PLR (IQR)	152.38 (120.8–191.34)	109 (88.96–138.02)	<0.001
Sedimentation (mm/h) (IQR)	24 (12–43)	14 (7–22.5)	<0.001
CRP (mg/L) (IQR)	10 (3–31)	4 (1–8)	<0.001

N: sample size, MCV: mean corpuscular volume, MCH: mean cell hemoglobin, MCHC: mean cell hemoglobin concentration, MPV: mean platelet volume, NLR: neutrophil-lymphocyte ratio, MLR: monocyte-lymphocyte ratio, PLR: platelet-lymphocyte ratio, WBC: white blood cell, P: significance level; PDW: platelet distribution width, RDW-CV: red cell distribution width coefficient of variation.

Similar to NLR, both MLR and PLR increased significantly (all P < 0.001) in EN compared to control group in secondary EN compared to idiopathic EN. 

We also showed NLR, MLR, and PLR to be correlated with each other and with other conventional inflammatory markers (Table 7). 

**Table 7:** Correlations between NLR, MLR, PLR and conventional inflammatory markers.

	NLR		MLR		PLR	
	r	P	r	P	r	P
WBC	0.38	<0.001	0.24	<0.001	–0.07	0.051
MPV	–0.25	<0.001	–0.14	<0.001	–0.28	< 0.001
PDW	0.27	<0.001	0.09	0.01	0.011	0.76
RDW-CV	0.17	<0.001	0.08	0.035	0.16	<0.001
Sedimentation	0.33	<0.001	0.19	<0.001	0.37	<0.001
CRP	0.44	<0.001	0.36	<0.001	0.31	<0.001
ASO	0.19	0.011	0.18	0.02	0.21	0.01
NLR	-	-	0.63	<0.001	0.59	<0.001
MLR	0.63	<0.001	-	-	0.44	<0.001
PLR	0.59	<0.001	0.44	<0.001	-	-

MPV: mean platelet volume, NLR: neutrophil-lymphocyte ratio, MLR: monocyte-lymphocyte ratio, PLR: platelet-lymphocyte ratio, WBC: white blood cell, P: significance level, PDW: platelet distribution width, RDWCV: red cell distribution width coefficient of variation, CRP: C reactive protein, ASO: antistreptolysin O.

### 3.3. Inflammatory markers as predictors of secondary EN

**Figure 4:**
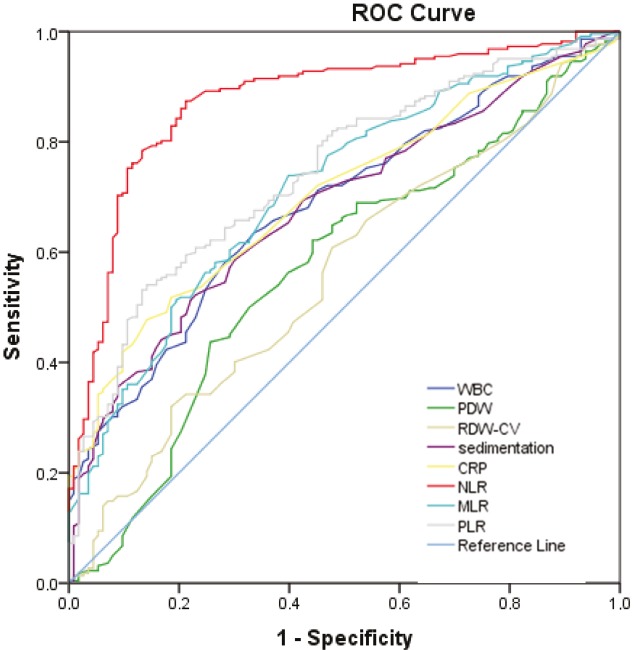
Receiver operating characteristic (ROC) curve analysis to identify predictors of secondary EN.

NLR, MLR, PLR, WBC, sedimentation, CRP, PDW, and RDW-CV were all increased in secondary EN patients compared to idiopathic EN. We performed receiver operating characteristic (ROC) curve analysis to identify predictors of secondary EN and their optimal cut-offs. The strongest predictor of secondary EN was NLR with an area under the curve (AUC) of 0.875 (Figure 4). The optimum cut-off for NLR was 2.11. NLR > 2.11 predicted secondary EN with 83.8% sensitivity and 80.5% specificity (P < 0.001). AUC, cut-offs, sensitivity, specificity, and statistical significance of MLR, PLR, WBC, sedimentation, CRP, PDW, and RDW-CV in predicting secondary EN are summarized in Table 8.

**Table 8:** AUC, cut-offs, sensitivity, specificity, and statistical significance of MLR, PLR, WBC, sedimentation, CRP, PDW, and RDW-CV in predicting secondary EN.

	RR (95% CI)	P value
Risk factor for EN		
NLR (>2.11)	17.88 (11.7–27.33)	<0.001
RDW-CV (>13.65)	3.06 (2.25–4.17)	<0.001
Risk factors for secondary EN		
NLR (>2.11)	17.16 (9.33–31.55)	<0.001
Recurrent EN	3.55 (1.49–8.49)	0.004
RDW-CV (>13.65)	2.69 (1.5–4.84)	0.001
CRP (>5.5)	2 (1.15–3.65)	0.014
Risk factors for recurrent EN		
Secondary EN	3.68 (1.78–7.61)	<0.001
Age (>40)	2.23 (1.27–3.94)	0.006
MCHC (<33)	2.39 (1.29–4.45)	0.006

NLR: neutrophil-lymphocyte ratio, MLR: monocyte-lymphocyte ratio, PLR: platelet-lymphocyte ratio, WBC: white blood cell, PDW: platelet distribution width, RDW-CV: red cell distribution width coefficient of variation, CRP: C reactive protein.

### 3.4. Multivariate logistic regression analysis 

Separate multivariate logistic regression analysis was performed to identify risk factors for EN, secondary EN, and recurrent EN. The values of NLR > 2.11 and RDW-CV > 13.65 increased the risk of EN (relative risks 17.88 and 3, respectively). Among EN patients, NLR > 2.11, recurrent EN, RDW-CV > 13.65, and CRP > 5.5 were risk factors for secondary EN (relative risks 17.16, 3.55, 2.69, and 2, respectively) (Table 9). Secondary EN, age > 40, and MCHC < 33 were risk factors for recurrent EN (relative risks 3.68, 2.23, and 2.39, respectively).

**Table 9:** Multivariate logistic regression analysis identifying risk factors for EN and secondary EN.

	Cut-off value	Sensitivity	Specificity	P value	AUC
WBC	7.95	64.4	64.6	<0.001	0.686
PDW	15.75	59.9	55.8	0.029	0.573
RDW-CV	13.65	60.8	52.2	0.068	0.561
Sedimentation	16.5	65.3	60.2	<0.001	0.683
CRP	5.5	67.1	60.2	<0.001	0.703
NLR	2.11	83.8	80.5	<0.001	0.875
MLR	0.24	68.5	62.8	<0.001	0.715
PLR	127.59	68	64.6	<0.001	0.744

NLR: neutrophil-lymphocyte ratio, RDW-CV: red cell distribution width coefficient of variation, CRP: C reactive protein, MCHC: mean cell hemoglobin concentration.

## 4. Discussion

EN is an inflammatory disorder of subcutaneous tissue characterized by erythematous, tender subcutaneous nodules predominantly affecting lower extremities. Although many etiologic factors such as infections, systemic disease, and drugs have been identified as causes of EN, many cases of EN still remain idiopathic. 

To our knowledge, there is no definitive clinical or laboratory data that can distinguish idiopathic EN from secondary EN. Our purpose in this study was to compare NLR levels in EN patients versus controls and evaluate NLR changes in the presence of a precipitating cause of EN. NLR was elevated in EN patients compared to controls. Among EN patients, NLR was also elevated in patients with secondary EN compared to idiopathic EN. 

Similar to figures reported in the literature, 34.7% of EN was idiopathic in our series (5,21,22). Infections (especially upper respiratory tract and urinary tract) and systemic diseases (especially rheumatologic diseases) were the leading causes of secondary EN. Although most of the patients with infection and systemic disease, which were listed as precipitating factors in patients’ files, were symptomatic, 41.5% of 82 EN patients with URTI were diagnosed by screening tests and microbiological workup after admission. Asymptomatic URTI, such as tonsillitis or pharyngitis, was diagnosed in 7% of 129 EN patients evaluated by Cribier et al. (6). The ratio of asymptomatic/symptomatic infection was much higher in our patients with UTI, the second most common infection among EN patients. Forty-six of 48 patients with UTI (95.8%) were diagnosed after urinary analysis and culture. These findings show us that patients with infections may be asymptomatic or may be showing mild symptoms, so laboratory and microbiological tests are essential to identify a possible infection as the etiological factor. The higher NLR shown in asymptomatic EN patients with infections makes this marker a candidate to prioritize the patients to be screened.

Systemic diseases were the second most common etiological factor of EN, and rheumatologic diseases were the most common systemic disease. Twelve of 71 patients (16.9%) had a history of rheumatologic disease, but in 59 patients (83.1%) the rheumatologic disease was diagnosed after EN. Similar to infections, patients with systemic diseases, both previously diagnosed and undiagnosed, had a higher NLR compared to patients with idiopathic EN. NLR may be a good candidate for a screening test for undiagnosed or asymptomatic infections and systemic diseases, and patients with high NLR should be investigated more carefully for an undiagnosed precipitating factor.

Our study showed that NLR increased in both idiopathic EN and secondary EN, but the increase in secondary EN was more prominent and significant. Systemic diseases and infection were the most frequent causes of secondary EN; previous studies revealed that NLR increases as an inflammatory marker in many systemic diseases (10–17). In our study, the more prominent and significant increase in NLR may be attributed to the secondary systemic and infectious diseases in EN. That’s why a more prominent increase in NLR should alert the physician to an underlying precipitating disease. 

Recurrence was identified in 16.2 % of EN patients. Advanced age, identification of an etiological factor, and low MCHC increased the risk of recurrent EN in multivariate logistic regression analysis. Papagrigoraki et al. also investigated the characteristics of relapsing EN. An etiological factor such as infection, drugs, systemic diseases, or pregnancy was identified in 75.8% of EN patients. Although infections, drugs, and pregnancy were more common in relapsing EN, multiple regression analysis showed that only drugs increase the risk of EN relapses (9). In our study, drug history was positive in 4.3% of EN patients and OCs was the most common drug, similar to other series in the literature (1,4,5,8,23). Identification and removal of the causes, especially drugs, are important for treatment of EN as well as for limiting the recurrence. 

Secondary EN was identified in 65.3% of patients. Patients with secondary EN were younger and had higher inflammatory markers than patients with idiopathic EN. After multivariate regression analysis, our results showed that high NLR (> 2.11), RDW-CV (> 13.65), CRP (> 5.5), and recurrence of EN predict secondary EN. Previous studies aimed to predict EN with an underlying precipitating factor, but none of the clinical or laboratory features investigated could predict secondary EN with high sensitivity and specificity. Dogan et al. investigated clinical and laboratory features of EN patients with an underlying systemic disease (24). The study showed that patients with complicated EN had higher platelet levels, and EN lesions were located at nonclassic localizations. Kisacik et al. reported that patients with secondary EN were younger and had higher sedimentation and CRP levels (25). Ozbagcivan et al. also investigated etiological factors in EN and they divided EN patients into 3 groups as idiopathic, infectious, and noninfectious EN (26). They compared both clinical and laboratory features of idiopathic, infectious, and noninfectious EN. Patients with infectious EN had fewer EN lesions, lower percentage of systemic symptoms, and higher ESR compared to those with noninfectious and idiopathic EN. On the other hand, patients with noninfectious EN had higher AST levels (26). ESR and CRP are well known inflammatory markers that are used in daily practice to identify infections and systemic diseases in EN patients. Our study showed that NLR was more sensitive and specific then previously studied ERS and CRP levels in identifying secondary EN. Although all 3 studies identified features of secondary EN, the predictive value of these features for secondary EN was not quantified. 

To the best of our knowledge this is the first study investigating NLR in EN patients. NLR was investigated as a novel inflammatory marker in many dermatological diseases. In psoriasis, NLR was increased compared to controls, decreased with treatment, and correlated with disease severity and conventional inflammatory markers (14,27,28). High NLR may also be used as a predictor of arthritis in psoriatic patients (29). NLR also increases in other dermatological diseases such as Behçet’s disease, liken planus, Hidradenitis Supurativa, vitiligo, and atopic dermatitis. Like in psoriasis, NLR correlates with disease severity and other inflammatory markers in these inflammatory skin diseases (15,30–34). 

Our study revealed that with a cutoff point of 2.11, NLR predicts EN among all participants and secondary EN among all EN patients with high sensitivity and specificity. The diagnostic value of NLR has been previously investigated in pneumonia and brucellosis. Yoon et al. showed that NLR with a cutoff point of 7 may distinguish between bacterial pneumonia and tuberculosis, and NLR < 7 is predictive for tuberculosis (35). Diagnosis of EN is based on clinical or histopathological features and NLR is an inflammatory marker which may increase in all inflammatory and neoplastic diseases. Using NLR for diagnosis of EN may therefore not be practical. However, our findings strongly suggest that selecting out secondary EN among clinically or histopathologically diagnosed EN patients with this cheap and easy marker is possible. 

This study was designed as a retrospective cross sectional study. Although we were able to retrospectively evaluate the medical records of a large group of EN patients, information on the clinical course of the disease was limited and we relied on existing information in patient charts. This precluded us from investigating the relationship between additional clinical characteristics such as atypical presentation and lesion count in predicting secondary EN. 

Identification and elimination of a possible underlying cause of EN is a very important step in treatment and not letting the precipitating factor go undiagnosed is important to limit recurrence. Medical history and physical examination are essential in identifying the cause, but in patients with previously undiagnosed comorbidities or asymptomatic infections additional laboratory work-up may be required. NLR is a cheap and easy marker that can be used to predict secondary EN with high sensitivity and specificity. High NLR can alert the physician against secondary EN, where extensive screening for a precipitating factor should not be neglected.

## References

[ref0] (2001). Mostly septal panniculitis. Journal of the American Academy of Dermatology.

[ref1] (2007). Sweet’s syndrome and erythema nodosum: two neutrophilic dermatoses?. Clinical Rheumatology.

[ref2] (1999). Leucocyte activation in erythema nodosum. Clinical and Experimental Dermatology.

[ref3] (2001). Erythema nodosum in children: a prospective study. Journal of the American Academy of Dermatology.

[ref4] (2004). Erythema nodosum: an experience of 10 years. Scandinavian Journal of Infectious Diseases.

[ref5] (1998). Erythema nodosum and associated diseases. A study of 129 cases. International Journal of Dermatology.

[ref6] (2008). Erythema nodosum. Dermatologic Clinics.

[ref7] (1992). Erythema nodosum. American Family Physician.

[ref8] (2010). Erythema nodosum: etiological factors and relapses in a retrospective cohort study. European Journal of Dermatology.

[ref9] (2001). Ratio of neutrophil to lymphocyte counts – rapid and simple parameter of systemic inflammation and stress in critically ill. Bratislavske Lekarske Listy.

[ref10] (2008). Association between admission neutrophil to lymphocyte ratio and outcomes in patients with acute coronary syndrome. American Journal of Cardiology.

[ref11] (2005). Neutrophil-lymphocyte ratio as a prognostic factor in colorectal cancer. Journal of Surgical Oncology.

[ref12] (2012). Neutrophil lymphocyte ratio as a measure of systemic inflammation in prevalent chronic diseases in Asian population. International Archives of Medicine.

[ref13] (2014). Neutrophil to lymphocyte ratio as a measure of systemic inflammation in psoriasis. Cutaneous and Ocular Toxicology.

[ref14] (2017). Assessment of neutrophil-to-lymphocyte ratio and platelet-to-lymphocyte ratio in atopic dermatitis patients. Medical Science Monitor.

[ref15] (2011). White blood cell count and stable coronary artery disease: the role of neutrophil to lymphocyte ratio. Cardiology Journal.

[ref16] (2011). Neutrophil-lymphocyte ratio as a predictor of adverse outcomes of acute pancreatitis. Pancreatology.

[ref17] (2011). Neutrophil/lymphocyte ratio predicts chemotherapy outcomes in patients with advanced colorectal cancer. British Journal of Cancer.

[ref18] (2011). Effect of preoperative neutrophil-lymphocyte ratio on the surgical outcomes of stage II colon cancer patients who do not receive adjuvant chemotherapy. International Journal of Colorectal Disease.

[ref19] (2010). High blood neutrophil-to-lymphocyte ratio is an indicator of poor prognosis in malignant mesothelioma patients undergoing systemic therapy. Clinical Cancer Research.

[ref20] (2000). Erythema nodosum: etiologic and predictive factors in a defined population. Arthritis & Rheumatology.

[ref21] (2000). Erythema nodosum in Singapore. Clinical and Experimental Dermatology.

[ref22] (1997). Development of erythema nodosum in the course of oestrogen replacement therapy. British Journal of Dermatology.

[ref23] (2016). Clinical and laboratory characteristics of patients with erythema nodosum. Skinmed.

[ref24] (2013). Multiclinical experiences in erythema nodosum: rheumatology clinics versus dermatology and infection diseases clinics. Rheumatology International.

[ref25] (2017). Examination of the microbial spectrum in the etiology of erythema nodosum: A retrospective descriptive study. Journal of Immunology Research.

[ref26] (2017). Neutrophil-lymphocyte ratio, platelet-lymphocyte ratio and mean platelet volume in Japanese patients with psoriasis and psoriatic arthritis: Response to therapy with biologics. Journal of Dermatology.

[ref27] (2017). Impact of elevated serum uric acid levels on systemic inflammation in patients with psoriasis. Angiology.

[ref28] (2016). Assessments of neutrophil to lymphocyte ratio and platelet to lymphocyte ratio in Korean patients with psoriasis vulgaris and psoriatic arthritis. Journal of Dermatology.

[ref29] (2015). The relation of neutrophil-to-lymphocyte ratio, platelet-to-lymphocyte ratio, and mean platelet volume with the presence and severity of Behcet’s syndrome. Kaohsiung Journal of Medical Sciences.

[ref30] (2016). Novel markers of endothelial dysfunction and inflammation in Behçet’s disease patients with ocular involvement: epicardial fat thickness, carotid intima media thickness, serum ADMA level, and neutrophil-to-lymphocyte ratio. Clinical Rheumatology.

[ref31] (2015). Patients with hidradenitis suppurativa carry a higher systemic inflammatory load than other dermatological patients. Archives of Dermatological Research.

[ref32] (2016). Assessment of systemic inflammation with neutrophil-lymphocyte ratio in lichen planus. Postępy Dermatologii i Alergologii.

[ref33] (2017). Neutrophil to lymphocyte ratio in patients with vitiligo. Postępy Dermatologii i Alergologii.

